# Research Priorities for Mental Health and Psychosocial Support in Humanitarian Settings

**DOI:** 10.1371/journal.pmed.1001096

**Published:** 2011-09-20

**Authors:** Wietse A. Tol, Vikram Patel, Mark Tomlinson, Florence Baingana, Ananda Galappatti, Catherine Panter-Brick, Derrick Silove, Egbert Sondorp, Michael Wessells, Mark van Ommeren

**Affiliations:** 1 Global Health Initiative, MacMillan Center, Yale University, New Haven, Connecticut, United States of America; 2 HealthNet TPO, Amsterdam, The Netherlands; 3 Sangath, Goa, India; 4 London School of Hygiene & Tropical Medicine, London, United Kingdom; 5 Department of Psychology, Stellenbosch University, Stellenbosch, South Africa; 6 Makerere University School of Public Health, Kampala, Uganda, and Personal Social Services Research Unit, London School of Economics and Political Science, London, United Kingdom; 7 Good Practice Group, Colombo, Sri Lanka; 8 Colombo University, Colombo, Sri Lanka; 9 Jackson Institute for Global Affairs and Department of Anthropology, Yale University, New Haven, Connecticut, United States of America; 10 School of Psychiatry, University of New South Wales, Sydney, Australia; 11 Program on Forced Migration and Health, Columbia University, New York, New York, United States of America; 12 Department of Mental Health and Substance Abuse, World Health Organization, Geneva, Switzerland

## Abstract

Wietse Tol and colleagues lay out a a consensus-based research agenda for mental health and psychosocial support in humanitarian settings.

**Summary Points**

- There has been a great need to develop a research agenda to strengthen mental health and psychosocial support in humanitarian settings; prior research in this area has had limited inputs from practitioners.
- We developed a consensus-based research agenda for the next ten years through inputs from an interdisciplinary group of academics, policy makers, and practitioners (n = 82) representing regions where humanitarian crises occur.
- Participants reached a high level of agreement on the ten most highly prioritized research questions, which consisted of questions related to: *problem analysis* (four questions on identifying stressors, problems, and protective factors from the perspective of affected populations), *mental health and psychosocial support interventions* (three questions on sociocultural adaptation and on effectiveness of family- and school-based prevention), *research and information management* (two questions on assessment methods and indicators for monitoring and evaluation), and *mental health and psychosocial support context* (one question on whether interventions address locally perceived needs).
- This research agenda emphasizes the generation of practical knowledge that could translate to immediate tangible benefits for programming in humanitarian settings, rather than addressing the key debates that have dominated the academic literature.
- Addressing this research agenda requires a better alignment between researchers and practitioners, attention to perspectives of populations affected by humanitarian crises, and sensitivity to sociocultural context.

## Introduction

In 2009, more than 119 million people were affected by natural disasters [1], and 36 armed conflicts were recorded in 26 countries [2]. Research in such settings has demonstrated the negative impact of humanitarian crises on mental health and psychosocial well-being, including increased psychological distress [3], social problems [4],[5], common mental disorders (depression and anxiety, including post-traumatic stress disorder [PTSD]), and severe mental disorders (e.g., psychotic disorders) [6].

Recent international policy [7],[8] indicates a growing consensus in the approaches recommended for mental health and psychosocial support interventions in humanitarian settings, despite a weak empirical evidence base to support specific approaches [9],[10]. Amongst both researchers and practitioners, however, divisions remain on key issues, notably (a) the extent to which PTSD should be a central research and intervention focus [11], (b) the distinction between normal psychological distress and mental disorders in situations of adversity [12], and (c) the extent to which interventions should aim to target mental disorders or ongoing structural and situational stressors in the recovery environment [9],[10],[13]. In addition, (d) practitioners and researchers have been divided over the extent to which research has led to tangible benefits for implementing programs, and about the universality of applied constructs of mental disorder [14]. A consensus-based research agenda for this area of work does not exist. Furthermore, the fact that the power to set the research agenda typically is vested in researchers from outside of humanitarian settings has the potential to marginalize many practitioners. Currently prioritized research may therefore not improve the knowledge that is needed by practitioners on the ground [15].

The Mental Health and Psychosocial Support in Humanitarian Settings – Research Priority Setting (MH-SET) project was initiated to establish a consensus-based research agenda aimed at supporting the prevention and treatment of mental disorders and the protection and promotion of psychosocial well-being in humanitarian settings. The project aimed to set research priorities based on the perspectives of a range of key stakeholders, including academics, practitioners, and policy makers from a variety of disciplines, ensuring representation from locations where humanitarian crises occur. This report lays out the results of the, to our knowledge, first systematic effort to set research priorities in this field.

## Methods

**Figure 1:**
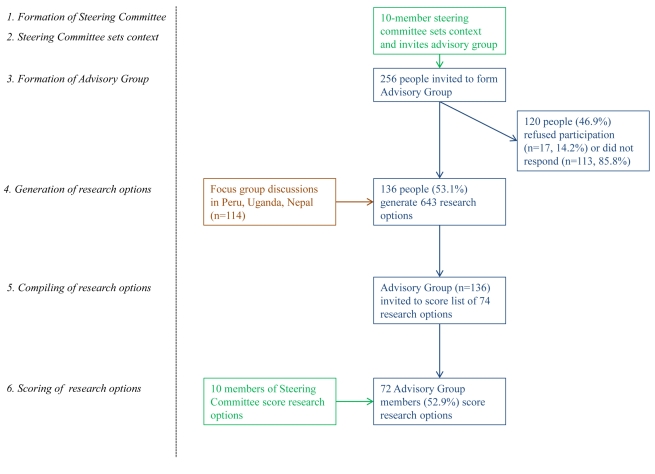
Research priority setting flow chart.

The MH-SET initiative adopted the widely implemented consensus-building methodology developed by the Child Health and Nutrition Research Initiative (CHNRI) (methods are described in more detail in Text S1). In brief, this method allows for the systematic generation and scoring of research questions using predetermined criteria (Figure 1). We selected this method because it allows for a structured approach to research priority setting, and because it defines research as an activity that aims to improve the lives of people rather than focusing on the generation of new knowledge per se. The CHNRI methodology has been used to set research priorities in a variety of fields, including child health, health of people with disabilities, zinc-related health research, mental health, preterm birth, stillbirth, and birth asphyxia [16]–[22].

### Stage 1: Defining the Research Context

An international steering committee (authors; 40% from low- and middle-income countries [LMIC]) decided to address global research priorities, focus on child and adult populations, and look broadly at mental disorders and psychosocial issues. In addition, we focused on setting priorities for questions that could be answered within the coming ten years.

### Stages 2–4: Formation of Advisory Group, Generation and Compilation of Research Questions

Research questions were generated by 136 advisory group members, and participants of nine focus group discussions in Peru, Uganda, and Nepal (n = 114). The advisory group generated research questions through an online survey format, where participants were invited to list up to five research questions they felt were crucial to support mental health and psychosocial support in humanitarian settings. Advisory group members in this step were 43.3% female, and worked in a variety of settings (academia 13.4%; implementation 38.8%; both 47.8%). Most members focused on both mental disorders and psychosocial well-being (65.4%; only mental disorders 3.7%; only psychosocial well-being 29.9%). Members of the advisory group were broadly representative of locations where humanitarian settings occur. The focus group discussions are described in more detail elsewhere [14]. Altogether, 733 research questions were generated, which were consolidated into a list of 74 research questions through qualitative data analysis by two independent analysts. This list contained research questions grouped into four categories: (1) Problem Analysis (14 research questions), (2) Mental Health and Psychosocial Support Interventions (25 research questions), (3) Mental Health and Psychosocial Support Context (25 research questions), (4) Research and Information Management (ten questions).

### Stage 5: Scoring Research Questions

Research questions were scored by (a) 53% of the advisory group that generated research options (n = 72) and (b) the ten members of the steering committee (authors) using five predetermined criteria related to significance, answerability, applicability, equity, and ethics (Box 1). Scoring was conducted through an online survey format, with follow-up reminders by email. We assessed whether the 72 respondents in this step were different from those participating in the generation of research options, and did not find any differences with regard to gender, region of work/origin/residence, affiliation, implementation focus, or work setting (Chi-square comparisons; p-values 0.11–0.79). Final endorsement scores were calculated by taking the overall average of the average endorsement (Yes = 1, No = 0) for each of the five research criteria.

**Box 1. Criteria Used to Score Research Questions**

**Criterion 1. Significance**

Would you say the research question is an important question that needs answering? In other words, do you think this research question is essential to address in the coming 10 years? Please rate as “0. No” (Not at all important) or “1. Yes” (Essential).

**Criterion 2. Answerability**

Would you say that a study to answer this question is feasible? In other words, do you think it is possible to actually design a study that addresses this research question in the coming 10 years? Please rate as “0. No” (Not at all feasible) or “1. Yes” (Very feasible).

**Criterion 3. Applicability**

Would you say that an answer to this research question would influence humanitarian policy and practice? In other words, do you think answering this research question will lead to tangible practice results in the coming 10 years? Please rate as “0. No” (Not at all applicable) or “1. Yes” (Very applicable).

**Criterion 4. Equity**

Would you say that an answer to this question would help to improve the conditions of marginalized groups in humanitarian settings? In other words, do you think answering this research question will aid underprivileged populations in the coming 10 years? Please rate as “0. No” (Does not improve) or “1. Yes” (Will very much improve).

**Criterion 5. Ethics**

Would you say that a study to answer this question would be perceived as ethical by all of the key stakeholder groups (e.g. affected population, national governments, humanitarian agencies, donors)? In other words, do you think this question may be answered in an ethical manner in the coming 10 years? Please rate as “0. No” (Not ethical) or “1. Yes” (Ethical).

## Results

Table 1 lists the ten most highly prioritized research questions. These ten research questions were rated with high agreement by participants; all of the research questions scored above 80% average endorsement as “essential” on each of the five research criteria. Overall, the top ten appears to emphasize a research agenda for the next ten years focusing on research questions that may have immediate benefit for humanitarian programs, rather than addressing the key debates that have dominated the academic literature (e.g. the controversy surrounding PTSD, the distinction between distress and disorder). Specific categories of research questions that feature prominently in the ten most highly prioritized research questions are (in order of importance): (1) Problem Analysis (four research questions), (2) Mental Health and Psychosocial Support Interventions (three questions), (3) Research and Information Management (two questions), and (4) Mental Health and Psychosocial Support Context (one question).

**Table 1:** Ten most highly endorsed research questions.

Research Option	Category	Average Rating (%)
1. What are the stressors faced by populations in humanitarian settings?	Problem Analysis	86.7
2. What are appropriate methods to assess mental health and psychosocial needs of populations in humanitarian settings?	Research and Information Management	85.9
3. How do affected populations themselves describe and perceive mental health and psychosocial problems in humanitarian settings?	Problem Analysis	85.9
4. What are appropriate indicators to use when monitoring and evaluating the results of mental health and psychosocial support in humanitarian settings?	Research and Information Management	85.4
5. How can we best adapt existing mental health and psychosocial interventions to different sociocultural settings?	MHPSS Interventions	85.2
6. What is the effectiveness of family-based interventions to prevent mental disorders and protect and promote psychosocial well-being and mental health among children and adolescents in humanitarian settings?	MHPSS Interventions	84.7
7. What are the major protective factors (including individual [e.g., coping, hope] and contextual [e.g., justice mechanisms, religious practices]) for mental health and psychosocial problems in humanitarian settings?	Problem Analysis	84.4
8. What is the effectiveness of school-based psychosocial and mental health interventions to prevent mental disorders and protect and promote psychosocial well-being and mental health among children and adolescent in humanitarian settings?	MHPSS Interventions	83.2
9. To what extent do current mental health and psychosocial supports address locally perceived needs?	MHPSS Context	82.5
10. Which are the most common mental health and psychosocial problems in the general population in humanitarian settings?	Problem Analysis	82.2

MHPSS, Mental Health and Psychosocial Support.

### 1. Problem Analysis

Research questions in this category concerned the stressors faced by populations in humanitarian settings (#1), local perceptions of the mental health and psychosocial impact of humanitarian crises (#3), major protective factors for mental health and psychosocial problems (#7), and the most common mental health and psychosocial problems in the general population in humanitarian settings (#10). Rather than assume the importance of specific mental disorders or psychosocial constructs, the prioritization of these questions seems to favor a research agenda that takes a step back and examines the most important stressors, problems, and protective factors from the perspective of affected populations.

### 2. Mental Health and Psychosocial Support Interventions

In this category, participants prioritized research that facilitates the adaptation of existing interventions to different sociocultural settings (#5) and that evaluates the effectiveness of family- and school-based interventions to prevent mental disorders and promote and protect psychosocial well-being in humanitarian settings (#6 and #8, respectively). In addition to addressing the importance of attention to the differing sociocultural contexts in which humanitarian settings occur, these questions call specifically for more research on prevention and promotion.

### 3. Research and Information Management

Two questions in this category were prioritized—one question on assessment methods (#2) and one on the selection of indicators for monitoring and evaluation (#4). Again, these research questions appear to support an agenda that is focused on improving practice and that supports a fresh look at how to measure the impact and changes over time of mental health and psychosocial well-being in humanitarian crises.

### 4. Mental Health and Psychosocial Support Context

Finally, one prioritized question from this category focused on whether current interventions address locally perceived needs (#9). Similar to questions #3 and #5, this question highlights the importance of considering local perspectives on the appropriate methods of addressing psychosocial and mental health problems in humanitarian settings.

## Discussion

The MH-SET initiative used established methods of research priority setting and incorporated the perspectives of academics, practitioners, and policy makers from a variety of disciplines working in geographically diverse humanitarian settings.

The findings offer a number of directions for further research and practice. First, the most highly prioritized research questions favored practical initiatives with a strong potential for translation of knowledge into mental health and psychosocial support programming. The tendency to emphasize research that informs practice is further suggested by the fact that the majority of research questions in the overall compilation were related to effectiveness and implementation (50 out of 74; 67.6%). To illustrate, as we note in the Introduction, controversy has surrounded the psychiatric diagnosis of PTSD. Despite the central role this debate has played in the literature, only a limited portion of the generated research questions were trauma-focused (42 out of the initially generated 733 questions [6%]). Based on this incongruity, we recommend a better alignment between academic priorities and those of practitioners. This may be stimulated through (a) strengthened partnerships between humanitarian agencies and universities, (b) upgrading basic research skills of humanitarian practitioners to strengthen information gathering as part of program implementation, and (c) increased funding for the research questions prioritized in this study.

Second, we note that three of the ten most highly prioritized research questions emphasize the inclusion of perspectives from affected people and the promotion of sensitivity to the sociocultural context. We recommend, in accordance with current international policy [7],[8], that researchers in humanitarian settings more strongly emphasize these aspects in their work. In our experience, prioritizing the strengthening of local research infrastructure in humanitarian settings—especially in low- and middle-income settings—as an integrated goal in research projects may form an important contribution to this end.

Third, we note the particular dominance (four out of the top ten questions) of problem analysis research. Although there is already a very large body of research that has focused on establishing prevalence rates of PTSD and depression in post-conflict and natural disaster settings [3],[23], the prioritized questions on problem analysis cover much broader ground, in that they concern major stressors faced, mental health and psychosocial problems as defined by populations affected by humanitarian crises, protective factors, and an open question on what the most common mental health and psychosocial problems in humanitarian settings are. The focus on problem analysis research may reflect the lack of systematic use of needs assessments in the design of mental health and psychosocial support programs [10],[24]. In accordance with the results of this study and recommendations in international policy [7],[8] we recommend a stronger emphasis on needs assessments as a structural element of practice.

Fourth, in relation to specific interventions, research into the effectiveness of family- and school-based preventive interventions scored highly. Research on children and adolescents was similarly highly rated in two previous research priority setting efforts in the general field of global mental health [18],[25].

### Study Limitations

We point to a number of limitations of this exercise. First, although we achieved targeted representation from four of eight regions, we had smaller than intended representation from two regions (Eastern Asia and the Pacific; Latin America and the Caribbean) and stronger representation than targeted from two regions (Middle East and North Africa; industrialized countries). The higher representation from industrialized countries may be due to the inclusion of global practitioners, who work in the countries where they reside as well as in other regions. We did, however, achieve a sample that consisted of two-thirds of participants originating in LMICs, and who worked in 47 different languages. Second, we had an attrition rate of 53% between the phases of generating and scoring research questions. Although we did not find any differences between responders and nonresponders on sociodemographic and occupational variables, the pattern of nonresponse may have contained other biases that were not measured.

## Conclusions

Our research priority setting initiative—the first of its kind in this particular field—showed promising points of agreement between diverse stakeholders on research priorities for mental health and psychosocial support in humanitarian settings. There was a strong endorsement of research that achieves tangible benefits for programming and that gives emphasis to participation with and sensitivity to the specific sociocultural context of the populations living in humanitarian settings.

## Supporting Information

Text S1 **Supplementary information.**
(DOC)

## Abbreviations

CHNRI: Child Health and Nutrition Research Initiative
LMIC: low- and middle-income country(ies)
MH-SET: Mental Health and Psychosocial Support in Humanitarian Settings – Research Priority Setting [project]
PTSD: post-traumatic stress disorder
