# Supplementary material for: Research Priorities for Mental Health and Psychosocial Support in Humanitarian Settings
Source: PLoS Med. 2011 Sep 20;8(9):e1001096. doi: 10.1371/journal.pmed.1001096 (PMC3176752; doi:10.1371/journal.pmed.1001096)
Supplement: Text S1 — Supplementary information. (DOC) [file pmed.1001096.s001.doc]

**Research Priorities for Mental Health and Psychosocial Support in Humanitarian Settings *– Supplementary Information***

Tol, Wietse A.^1^; Patel, Vikram^2^; Tomlinson, Mark^3^; Baingana, Florence^4^; Galappatti, Ananda^5^; Panter-Brick, Catherine^6^; Silove, Derrick^7^; Sondorp, Egbert^8^; Wessells, Michael^9^; van Ommeren, Mark^10^

^1^ Global Health Initiative, MacMillan Center, Yale University (New Haven, USA) & HealthNet TPO (Amsterdam, the Netherlands)

^2^ Sangath (Goa, India) & London School of Hygiene & Tropical Medicine (London, UK)

^3^ Department of Psychology, Stellenbosch University (Matieland, South Africa)

^4^ School of Public Health, Makerere University (Kampala, Uganda)

^5^Good Practice Group, Colombo & Colombo University (Colombo, Sri Lanka)

^6^ Jackson Institute for Global Affairs & Department of Anthropology, Yale University

^7^ School of Psychiatry, University of New South Wales (Sydney, Australia)

^8^ London School of Hygiene & Tropical Medicine (London, UK)

^9^ Mailman School of Public Health, Columbia University (New York, USA)

^10^ Department of Mental Health and Substance Abuse, World Health Organization (Geneva, Switzerland)

**Methods**

*Formation of an international steering committee and defining the context*

The authors of this paper constituted a small international steering committee in response to discussions on the need for establishing a consensus-based research agenda, initiated during two international meetings on mental health and psychosocial support in humanitarian settings, held in London during 2008. This international steering group was constituted with an eye on creating diversity in terms of (a) affiliation (academic and implementation institutions); (b) geographical focus; (c) implementation focus (biomedical and psychosocial orientations); and (d) discipline (medical, behavioral and social sciences). Our ten members included mental health professionals (n=4), public health practitioners (n=3), child protection/ psychosocial experts (n=2), and an anthropologist (n=1). Four members of the steering committee were from low- and middle-income countries.

The steering committee defined the context for setting research priorities, with regard to scale and time period (see Box 1 for an overview of the research priority setting process). We defined the focus on humanitarian crises in any part of the world (i.e. low-, middle, and high-income countries), and chose a time period of 10 years as an appropriate time horizon for a research agenda. In other words, we focused on setting a research agenda with questions that could be answered within the coming 10 years. With regard to the target population and problem focus, we chose to focus broadly on mental disorders and psychosocial wellbeing of people affected by humanitarian crises, in accordance with the terminology in the Inter-Agency Standing Committee (IASC) Guidelines

*Formation of an advisory group and generation of research options*

We identified a large advisory group to generate and score research options, as follows (Figure 1). First, we estimated the necessary size of the advisory group. Previous research priority setting using the CHNRI methodology has been conducted with samples ranging from 6 in the area of child health in South Africa ; 7 in zinc related health research ; 24 in mental health research to 50 in the area of disability and health . Aiming for at least 50 participants, and taking into account 60% attrition rates at both requests to the advisory group (i.e. generating and scoring research options), we aimed for an initial sample of around 250 participants. We purposively sampled for (a) a group of people with expertise in countries that would be representative of populations affected by humanitarian crises, and (b) a group of global experts, comprising around 75% and 25% of the advisory group respectively.

Second, we decided on appropriate representation of relevant geographical foci within the group with national expertise (i.e., the group constituting 75% of the advisory group). We listed all countries involved in appeals for funding in the United Nations Consolidated Appeals Process (from 2005 to 2009) and the Central Emergency Response Fund (from inception at March 1^st^ 2006 to 2009). We subsequently categorized these countries according to the 8 UNICEF regions of the world , and calculated what average percentage of appeals originated from these regions. To correct for population, we multiplied the percentages for regional populations by the regions percentage contribution to the world population, according to the July 2009 estimates of the Central Intelligence Agency World Factbook . This resulted in the following division according to regions (with 188 [75% of 250] as the denominator): East Asia and the Pacific, N=51 (27%); South Asia, N=55 (29%); Central and Eastern Europe and the Commonwealth of Independent States, N= 7 (4%); Middle East and North Africa, N=14 (7%); Eastern and Southern Africa, N=26 (14%); West and Central Africa, N=18 (10%); Latin America and the Caribbean, N=14 (8%); Industrialized countries, N=3 (1%).

Third, we drafted a list of potential members using predetermined criteria including (a) authors cited more than 50 times in a Google Scholar search (key words “armed conflict” or “disasters” and “mental health” or “psychosocial”); (b) first authors in key publications (books and review articles) ; (c) first authors on papers in the journal Intervention (a specialized journal with a wide readership in practice settings ); (d) members of the IASC Reference Group on Mental Health and Psychosocial Support in Emergencies (established in 2007 to disseminate and promote implementation of the IASC Guidelines ); (e) writers and reviewers of the IASC Guidelines, and (f) a subset of key contacts from members of the steering committee.

Fourth, we categorized the compiled list of possible advisory group members from step 3 according to these 8 world regions and a 9^th^ category of global experts, based upon the steering committee’s knowledge of the focus of potential advisory group members’ work and by searching the internet with a combination of potential members’ names and relevant key words.

Altogether, 256 individuals were invited by email with a request for each to generate 5 research options in an online questionnaire format using the website www.surveymonkey.com (“Which research questions do you feel are important to support mental health and psychosocial support in humanitarian settings?”), with respondents completing information on their socio-demographic characteristics, work setting (implementation, academic, or both), focus (mental disorders, psychosocial wellbeing, or both), and work setting. In addition, we recommended that advisory group members consult with colleagues in the generation of research options if they so wished. This optional consultation of colleagues was intended to stimulate the wider inclusion of perspectives from researchers, practitioners and grassroots workers in humanitarian settings.

*Focus Group Discussions*

Because we were interested in establishing research priorities with the inclusion of perspectives from humanitarian settings, we extended the method to conduct focus group discussions in Peru, Uganda, and Nepal. These settings were selected purposively to ensure geographical diversity and to sample for humanitarian settings related to both armed conflicts and natural disasters. Details of these focus group discussions are described elsewhere . In short, from August 2009 to February 2010 we conducted 9 focus group discussions with 114 participants (48% female) in both the capitals of countries and in locations of humanitarian crises (earthquake-affected area in Peru, conflict-affected area in Uganda, and flood- and conflict-affected areas in Nepal). Team leaders in each country purposively sampled participants to include key national stakeholders from the academic and program implementation community, representing a variety of disciplines (anthropology, psychology, psychiatry, public health) and work settings (government, diverse humanitarian sectors, including protection, education and health sectors). Members in each focus group were requested to achieve consensus on the ten leading priorities for research on mental health and psychosocial support in humanitarian settings .

We obtained informed consent both from the advisory group and the participants of focus group discussions, and research was conducted following the principles expressed in the Declaration of Helsinki .

*Scoring research options*

Research options generated by the advisory group and focus group discussions were compiled and categorized by two independently working data analysts (WT and an external analyst), who analyzed the text thematically. To reduce the risk of bias, these two analysts were unaware of the identities and affiliations of participants. Research questions that were generated by 1 participant only were excluded from analysis. Analysts separately (a) grouped all research questions into categories, and (b) merged duplicate questions. Agreement between the two analysts was reached on the main categories. Minor disagreements on sub-division of questions amongst categories were identified, discussed and resolved by the two analysts.

Advisory group members who participated in the generation of research options were requested to score this compiled list, using 5 research criteria. The steering committee pre-selected these criteria from a list used in previous studies, selecting criteria with the highest perceived relevance for the specific field of study. These criteria were: (a) *Significance* (‘Is the research question essential to answer in the coming 10 years?’), (b) *Answerability* (‘Is it possible to design a study that addresses this research question in the coming 10 years?’), (c) *Applicability* (‘Will answering the research question lead to tangible practice results in the coming 10 years?’), (d) *Equity* (‘Will answering the research question aid underprivileged populations in the coming 10 years?’), and (e) *Ethics* (‘Can the research question be answered in an ethical manner in the coming 10 years?’), and were answered in a yes/ no format. We did not provide this format in Spanish, as only 2 participants had filled out the Spanish questionnaire to generate research options (1 of whom subsequently filled out the English questionnaire). We used the resulting scores to calculate average endorsement per criteria, by summing up all scores and dividing these by amounts of ratings received for that research question. A final endorsement score per research option was calculated by taking the average over the 5 criteria.

Both the questionnaires for the generation and scoring of research options were piloted, and adjustments to language and formatting were made accordingly.

**Results**

*Advisory group and research options generated*

Out of the 256 people invited, 136 people generated research questions (53%). Demographic and occupational characteristics of this group are shown in Table 1. Almost half were women (43%), while 48% worked in both academic and implementation settings. Only 4% of the advisory group stated that their focus was on mental disorders only. Two-third reported a focus on both mental disorders and psychosocial wellbeing, while 30% focused on psychosocial wellbeing only.

With regard to geographical representation (Figure 2), the 4 common regions where advisory group members worked were South Asia (26%), East Asia and the Pacific (17%), Eastern and Southern Africa (16%), and the Middle East and North Africa (14%). Compared to our targeted distribution, we achieved (within 3 percentage points) representation from South Asia (26% achieved, 29% targeted), Central and Eastern Europe and the Commonwealth of Independent States (2% achieved, 4% targeted), Eastern and Southern Africa (16% achieved, 14% targeted), and West and Central Africa (7% achieved, 10% targeted). We did not achieve desired representation from Eastern Asia and the Pacific (17% achieved, 27% targeted) and Latin America and the Caribbean (6% achieved, 14% targeted), and we achieved larger than estimated participation from the Middle East and North Africa (14% achieved, 7% targeted) and Industrialized countries (12% achieved, 1% targeted).

Around a third of the advisory group reported originating from an industrialized country (34%), the other groups originating from South Asia (26%), East Asia and the Pacific (13%), Eastern and Southern Africa (10%), and Latin America and the Caribbean (7%). Altogether, advisory group members worked in 47 different languages (in order of frequency): English (41%), French (11%), Arabic (4%), Tamil (4%), Spanish (4%), Bahasa Indonesia (4%), Hindi (3%), and Sinhalese (3%).

371 people (136 advisory group members, 121 consulted colleagues, and 114 focus group discussion participants) generated 733 research questions (643 by the advisory group and colleagues, and 90 by the focus group participants).

*Research options priority scores*

All research options were compiled into a list of 74 research options, and the 136 members of the advisory group who generated research options then were invited to score this list. In addition, the international steering committee (n=10) scored the same list of research options. The final list consisted of research questions assigned to 4 categories in the following order; (1) *problem analysis* (14 research questions, e.g. questions related to which stressors and problems are common in humanitarian settings, and what risk and protective factors exist for mental health and psychosocial wellbeing in such settings for diverse population groups); (2) *mental health and psychosocial support interventions* (25 research questions, e.g. questions related to the effectiveness of diverse approaches); (3) *mental health and psychosocial support context* (25 research questions, e.g. questions related to the implementation of mental health and psychosocial support, including sustainability, training, financial, human resources and contextual issues); and (4) *research issues and information management* (10 research questions, e.g. questions related to assessment, screening/ diagnostic procedures, monitoring and evaluation).

In total, 72 advisory group members scored research options (53% of the advisory group that generated research options), using the 5 criteria. We assessed whether respondents in this step were different from those participating in the generation of research options. We did not find any statistically significant differences (chi-square comparisons) with regard to gender, region of work/ origin/ residence, affiliation, implementation focus or work setting (p values ranging from .11 to .79).

**References**

1. Inter-Agency Standing Committee [IASC] (2007) IASC Guidelines on Mental Health and Psychosocial Support in Emergency Settings. Geneva: IASC.

2. Tomlinson M, Chopra M, Sanders D, Bradshaw D, Hendricks M, et al. (2007) Setting priorities in child health research investments for South Africa. PLoS Med 4: e259.

3. Brown KH, Hess SY, Boy E, Gibson RS, Horton S, et al. (2009) Setting priorities for zinc-related health research to reduce children's disease burden worldwide: an application of the Child Health and Nutrition Research Initiative's research priority-setting method. Public Health Nutr 12: 389-396.

4. Tomlinson M, Rudan I, Saxena S, Swartz L, Tsai AC, et al. (2009) Setting priorities for global mental health research. Bulletin of the World Health Organization 87: 438-446.

5. Tomlinson M, Swartz L, Officer A, Chan KY, Rudan I, et al. (2009) Research priorities for health of people with disabilities: an expert opinion exercise. Lancet 374: 1857-1862.

6. UNICEF (2011) Information by Country. New York: UNICEF.

7. Central Intelligence Agency (2009) The World Factbook. Washington, DC: CIA.

8. Boothby N, Strang A, Wessells M (2006) A World Turned Upside Down: Social Ecological Approaches to Children in War Zones. Bloomfield, CT: Kumarian Press, Inc.

9. Miller KE, Rasco LM (2004) The Mental Health of Refugees: Ecological Approaches to Healing and Adaptation. Mahwah, NJ: Lawrence Erlbaum Associates.

10. Morris J, Van Ommeren M, Belfer M, Saxena S, Saraceno B (2007) Children and the Sphere standard on mental and social aspects of health. Disasters 31: 71-90.

11. Mollica RF, Cardozo BL, Osofsky HJ, Raphael B, Ager A, et al. (2004) Mental health in complex emergencies. Lancet 365: 842 - 843.

12. Batniji R, van Ommeren M, Saraceno B (2006) Mental and social health in disasters: relating qualitative social science research and the Sphere standard. Social Science & Medicine 62: 1853 - 1864.

13. Mollica RF, Guerra R, Bhasin R, Lavelle J (2004) Project 1 Billion Book of Best Practices: Trauma and the Role of Mental Health in Post-Conflict Recovery. Rome: Project 1 Billion: International Congress of Ministers of Health for Mental Health and Post-Conflict Recovery.

14. Patel V, Araya R, Chatterjee S, Chisholm D, Cohen A, et al. (2007) Treatment and prevention of mental disorders in low-income and middle-income countries. Lancet 370: 991-1005.

15. Hobfoll SE, Watson P, Bell CC, Bryant RA, Brymer MJ, et al. (2007) Five elements of immediate and mid-term mass trauma intervention: empirical evidence. Psychiatry: Interpersonal and Biological Processes 70: 283-315.

16. de Jong JTVM, editor (2002) Trauma, War, and Violence: Public Mental Health in Socio-Cultural Context. New York: Kluwer Academic/ Plenum Publishers.

17. Intervention: International Journal of Mental Health, Psychosocial Work and Counselling in Areas of Armed Conflict.

18. Tol WA, Patel V, Tomlinson M, Baingana F, Galappatti A, et al. (Under review) Relevance or Excellence? Setting Research Priorities for Mental Health and Psychosocial Support in Humanitarian Settings.

19. World Medical Association (2008) Declaration of Helsinki - Ethical Principles for Medical Research Involving Human Subjects.

*SI* Box 1. CHNRI Process

| **Stage 1: Defining the research context**  When: April – May 2009  How: group discussions and subsequent emails  Results:   - International steering committee (n=10 [present authors], 40% from LAMIC) was constituted to reflect diversity in terms of affiliation, geographical focus, implementation focus, and academic discipline (see Figure 1) - This committee defined the context of the research priority setting by selecting geographical focus (global), population (children and adults), and problem focus (mental health and psychosocial wellbeing in humanitarian settings). We focused on setting priorities for research questions that could be answered within the coming 10 years. |
| --- |
| **Stage 2: Formation of advisory group**  When: May – June 2009  How: group discussions, literature search, and email exchange  Results:   - Potential members for an advisory group (n=256) - tasked with generating and scoring research questions – were identified considering: (a) geographical representation of regions where humanitarian settings occur (countries appealing for humanitarian funding registered in two international databases between 2005 – 2009, corrected for population in these regions); (b) inclusion of those with national expertise (75%) and global experts (25%); and (c) identification of experts through literature searches using Google Scholar, first authors of key publications , first authors in a specialized journal with a wide readership in practice settings , writers, reviewers and members of the IASC Guidelines and its reference group |
| **Stage 3: Generation of research questions**  When: July 2009 – February 2010  How: email invitation to an online survey format; focus group discussions  Results:   - In total, 733 research questions were generated by (a) the advisory group members (N=136, 53% response rate, Table 1) and their consulted colleagues (N=121), and (b) focus group discussions organized in Peru, Uganda, and Nepal (N=9, 114 participants). Focus group discussions were organized specifically to ensure inclusion of perspectives from humanitarian settings. |
| **Stage 4: Consolidation of research questions and selection of research criteria**  When: March – June 2010  How: qualitative data analysis by two independent raters; email discussions  Results:   - A final list of 74 research questions covering 4 categories: (1) *problem analysis* (14 research questions, e.g. questions related to which stressors and problems are common in humanitarian settings, and what risk and protective factors exist for mental health and psychosocial wellbeing in such settings for diverse population groups); (2) *mental health and psychosocial support interventions* (25 research questions, e.g. questions related to the effectiveness of diverse approaches); (3) *mental health and psychosocial support context* (25 research questions, e.g. questions related to the implementation of mental health and psychosocial support, including sustainability, training, financial, human resources and contextual issues); and (4) *research issues and information management* (10 research questions, e.g. questions related to assessment, screening/ diagnostic procedures, monitoring and evaluation). - Five research criteria selected by the steering committee: (a) *Significance* (‘Is the research question essential to answer in the coming 10 years?’), (b) *Answerability* (‘Is it possible to design a study that addresses this research question in the coming 10 years?’), (c) *Applicability* (‘Will answering the research question lead to tangible practice results in the coming 10 years?’), (d) *Equity* (‘Will answering the research question aid underprivileged populations in the coming 10 years?’), and (e) *Ethics* (‘Can the research question be answered in an ethical manner in the coming 10 years?’) |
| **Stage 5: Scoring of research questions**  When: July– November 2010  How: All 136 members of the advisory group and the international steering committee are invited to score research options using the 5 research criteria in an online survey format, using a yes/ no format.  Results:   - 72 out of the 136 invited advisory group members (53%) scored research questions |
| **Stage 5: Analysis and write-up**  When: December 2010 – February 2011  How: An intermediate score on each of the five research criteria was calculated by averaging endorsements over all participants. A total research priority scores is calculated as the average of the five intermediate scores.  Results:   - Research priority scores for 74 research questions |

*Figure 1. Research priority setting flow chart*

*3. Formation of Advisory Group*

*4. Generation of research options*

*6. Scoring of research options*

*1. Formation of Steering Committee*

*2. Steering Committee sets context*

*5. Compiling of research options*

256 people invited to form Advisory Group

136 people (53.1%) generate 643 research options

120 people (46.9%) refused participation (n=17, 14.2%) or did not respond (n=113, 85.8%)

72 Advisory Group members (52.9%) score research options

10 members of Steering Committee score research options

Advisory Group (n=136) invited to score list of 74 research options

Focus group discussions in Peru, Uganda, Nepal (n=114)

10-member steering committee sets context and invites advisory group

*SI Figure 1. Geographical representation Advisory Group*


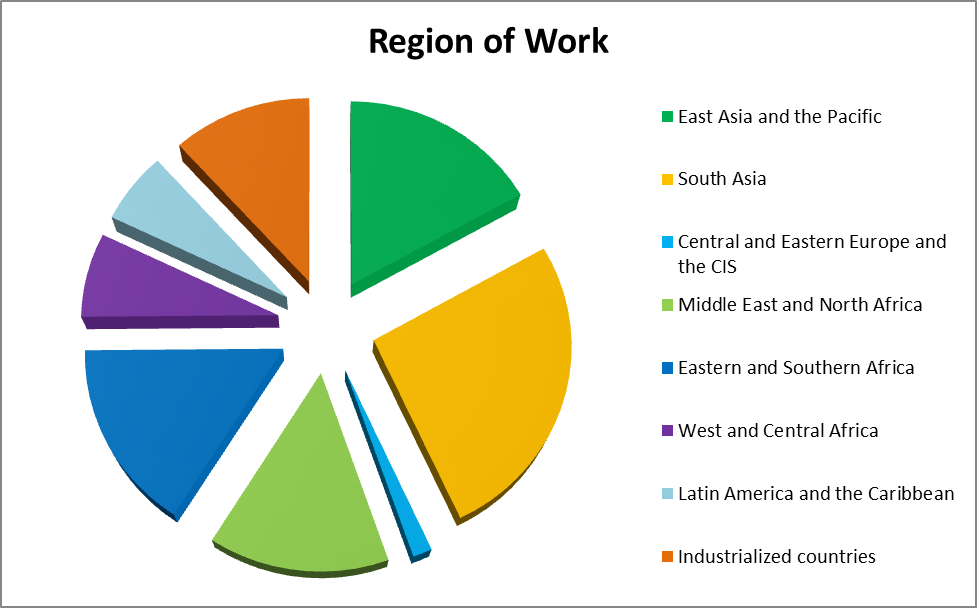


Note: CIS = Commonwealth of Independent States


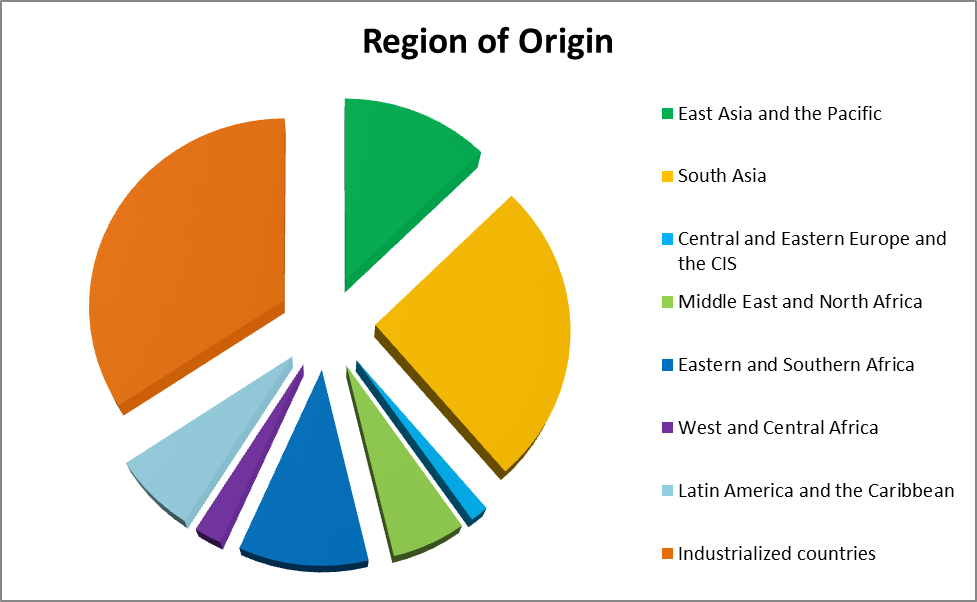


Note: CIS = Commonwealth of Independent States

*SI* Table 1. Characteristics Advisory Group (N=136)

|  |  | **N** | **%** |
| --- | --- | --- | --- |
| **Gender** | Female | 58 | 43.3 |
|  | Male | 76 | 56.7 |
| **Work setting** | Academic | 18 | 13.4 |
|  | Implementation | 52 | 38.8 |
|  | Both | 64 | 47.8 |
| **Work focus** | Mental disorders | 5 | 3.7 |
|  | Psychosocial wellbeing | 40 | 29.9 |
|  | Both | 89 | 65.4 |
| **Work place^a^** | International level | 71 | 53.0 |
|  | National level | 82 | 61.2 |
|  | Crisis location | 79 | 59.0 |

^a^ = multiple answers possible

*Table 1*. 10 most highly endorsed research options

| **Research Option** | **Category** | **Significance** | **Answerability** | **Applicability** | **Equity** | **Ethics** | **Average** |
| --- | --- | --- | --- | --- | --- | --- | --- |
| 1. What are the stressors faced by populations in humanitarian settings? | PA | 85.2 | 93.8 | 85.2 | 87.7 | 81.5 | 86.7 |
| 2. What are appropriate methods to assess mental health and psychosocial needs of populations in humanitarian settings? | RI | 88.9 | 82.7 | 86.4 | 85.2 | 86.4 | 85.9 |
| 3. How do affected populations themselves describe and perceive mental health and psychosocial problems in humanitarian settings? | PA | 88.9 | 86.4 | 80.2 | 86.4 | 87.7 | 85.9 |
| 4. What are appropriate indicators to use when monitoring and evaluating the results of mental health and psychosocial support in humanitarian settings? | RI | 93.8 | 75.3 | 87.7 | 82.7 | 87.7 | 85.4 |
| 5. How can we best adapt existing mental health and psychosocial interventions to different socio-cultural settings? | MI | 87.7 | 76.5 | 87.7 | 85.2 | 88.9 | 85.2 |
| 6. What is the effectiveness of family-based interventions to prevent mental disorders and protect and promote psychosocial wellbeing and mental health among children and adolescent in humanitarian settings? | MI | 96.3 | 80.2 | 80.2 | 85.2 | 81.5 | 84.7 |
| 7. What are the major protective factors (including individual [e.g. coping, hope] and contextual [justice mechanisms, religious practices]) for mental health and psychosocial problems in humanitarian settings? | PA | 90.1 | 77.8 | 84.0 | 82.7 | 87.7 | 84.4 |
| 8. What is the effectiveness of school-based psychosocial and mental health interventions to prevent mental disorders and protect and promote psychosocial wellbeing and mental health among children and adolescent in humanitarian settings? | MI | 88.9 | 77.8 | 86.4 | 84.0 | 79.0 | 83.2 |
| 9. To what extent do current mental health and psychosocial supports address locally perceived needs? | MC | 81.5 | 81.5 | 79.0 | 85.2 | 85.2 | 82.5 |
| 10. Which are the most common mental health and psychosocial problems in the general population in humanitarian settings? | PA | 79.0 | 87.7 | 82.7 | 81.5 | 80.2 | 82.2 |

Notes: PA = Problem Analysis; MI = Mental health and psychosocial support Interventions; MC = Mental health and psychosocial support Context; RI = Research Issues

*SI Table 2*. 10 least highly endorsed research options

| **Research Option** | **Category** | **Significance** | **Answerability** | **Applicability** | **Equity** | **Ethics** | **Average** |
| --- | --- | --- | --- | --- | --- | --- | --- |
| 65. How could technology (including internet, film, telephone) assist in the delivery of mental health and psychosocial support in humanitarian settings? | MC | 64.2 | 69.1 | 65.4 | 60.5 | 67.9 | 65.4 |
| 66. What is the effectiveness of psychosocial stimulation activities for young children to prevent mental disorders and protect and promote psychosocial wellbeing and mental health among young children in humanitarian settings? | MI | 65.4 | 61.7 | 61.7 | 64.2 | 67.9 | 64.2 |
| 67. What are effective ways to select staff for the delivery of mental health and psychosocial support in humanitarian settings? | MC | 69.1 | 59.3 | 63.0 | 59.3 | 69.1 | 64.0 |
| 68. How can we distinguish between 'normal' and 'pathological' mental health and psychosocial conditions in humanitarian settings? | RI | 65.4 | 61.7 | 61.7 | 65.4 | 64.2 | 63.7 |
| 69. How could a gender analysis assist in maximizing the impact of mental health and psychosocial support in humanitarian settings? | MC | 58.0 | 58.0 | 58.0 | 72.8 | 71.6 | 63.7 |
| 70. To what extent do disagreements in definitions of key concepts (e.g. psychosocial wellbeing, trauma) impact humanitarian practice? | RI | 56.8 | 56.8 | 60.5 | 63.0 | 71.6 | 61.7 |
| 71. What type of organizations are best positioned to deliver succesful mental health and psychosocial support in humanitarian settings? | MC | 64.2 | 49.4 | 59.3 | 63.0 | 61.7 | 59.5 |
| 72. What is the effectiveness of traditional and cultural healing practices (e.g. exorcism of evil spirits, rituals aimed at cleansing or forgiveness, meditation practices) to prevent mental disorders and protect and promote psychosocial wellbeing and mental health in humanitarian settings? | MI | 70.4 | 49.4 | 59.3 | 58.0 | 53.1 | 58.0 |
| 73. How do biological, psychological, and social factors interact in the long term to influence mental health and psychosocial wellbeing in humanitarian settings? | PA | 72.8 | 28.4 | 56.8 | 59.3 | 64.2 | 56.3 |
| 74. Are there universal definitions and diagnostic criteria for mental health and psychosocial problems in humanitarian settings? | RI | 46.9 | 45.7 | 43.2 | 46.9 | 54.3 | 47.4 |

Notes: PA = Problem Analysis; MI = Mental health and psychosocial support Interventions; MC = Mental health and psychosocial support Context; RI = Research Issues

*SI Table 3*. Research questions and their priority scores

| **Rank** | **Category** | **Question** | **Significance** | **Answerability** | **Applicability** | **Equity** | **Ethics** | **Average** |
| --- | --- | --- | --- | --- | --- | --- | --- | --- |
| 1 | PA | 1. What are the stressors faced by populations in humanitarian settings? | 85.2 | 93.8 | 85.2 | 87.7 | 81.5 | 86.7 |
| 2 | RI | 70. What are appropriate methods to assess mental health and psychosocial needs of populations in humanitarian settings? | 88.9 | 82.7 | 86.4 | 85.2 | 86.4 | 85.9 |
| 3 | PA | 11. How do affected populations themselves describe and perceive mental health and psychosocial problems in humanitarian settings? | 88.9 | 86.4 | 80.2 | 86.4 | 87.7 | 85.9 |
| 4 | RI | 71. What are appropriate indicators to use when monitoring and evaluating the results of mental health and psychosocial support in humanitarian settings? | 93.8 | 75.3 | 87.7 | 82.7 | 87.7 | 85.4 |
| 5 | MI | 14. How can we best adapt existing mental health and psychosocial interventions to different socio-cultural settings? | 87.7 | 76.5 | 87.7 | 85.2 | 88.9 | 85.2 |
| 6 | MI | 16. What is the effectiveness of family-based interventions to prevent mental disorders and protect and promote psychosocial wellbeing and mental health among children and adolescent in humanitarian settings? | 96.3 | 80.2 | 80.2 | 85.2 | 81.5 | 84.7 |
| 7 | PA | 7. What are the major protective factors (including individual [e.g. coping, hope] and contextual [justice mechanisms, religious practices]) for mental health and psychosocial problems in humanitarian settings? | 90.1 | 77.8 | 84.0 | 82.7 | 87.7 | 84.5 |
| 8 | MI | 17. What is the effectiveness of school-based psychosocial and mental health interventions to prevent mental disorders and protect and promote psychosocial wellbeing and mental health among children and adolescent in humanitarian settings? | 88.9 | 77.8 | 86.4 | 84.0 | 79.0 | 83.2 |
| 9 | MC | 47. To what extent do current mental health and psychosocial supports address locally perceived needs? | 81.5 | 81.5 | 79.0 | 85.2 | 85.2 | 82.5 |
| 10 | PA | 2. Which are the most common mental health and psychosocial problems in the general population in humanitarian settings? | 79.0 | 87.7 | 82.7 | 81.5 | 80.2 | 82.2 |
| 11 | MC | 62. What are the best ways to organize training and supervision of people delivering mental health and psychosocial support in humanitarian settings? | 90.1 | 80.2 | 81.5 | 76.5 | 81.5 | 82.0 |
| 12 | MC | 42. What are the social and economic impacts of mental health and psychosocial support in humanitarian settings? | 91.4 | 66.7 | 77.8 | 86.4 | 84.0 | 81.3 |
| 13 | PA | 13. How does the effectiveness of mental health and psychosocial support vary across specific populations groups (e.g. women, children, elderly, forced migrants, homeless children, people with disabilities, religious/ ethnic groups)? | 87.7 | 65.4 | 77.8 | 88.9 | 86.4 | 81.2 |
| 14 | PA | 15. How can we best develop existing mental health and psychosocial interventions within different socio-cultural settings? | 85.2 | 75.3 | 82.7 | 80.2 | 82.7 | 81.2 |
| 15 | MI | 36. What is the effectiveness of interventions aimed at decreasing the mental health and psychosocial burden of humanitarian staff? | 79.0 | 81.5 | 86.4 | 74.1 | 81.5 | 80.5 |
| 16 | MC | 50. What are existing strengths and capacities (e.g. community leadership, indigenous expertise, religious/ spiritual structures) of communities in humanitarian settings? | 84.0 | 74.1 | 81.5 | 79.0 | 82.7 | 80.3 |
| 17 | MC | 55. How can sustainability of mental health and psychosocial support in humanitarian settings best be maximized? | 88.9 | 63.0 | 80.2 | 84.0 | 82.7 | 79.8 |
| 18 | PA | 3. What are the most common mental health and psychosocial problems in specific population groups (e.g. women, children, elderly, forced migrants, homeless children, religious/ ethnic groups) in humanitarian settings? | 81.5 | 82.7 | 79.0 | 84.9 | 70.4 | 79.7 |
| 19 | PA | 5. What are the impacts of mental health and psychosocial problems for other outcomes in humanitarian settings (including economic, physical health, educational, and social outcomes)? | 88.9 | 59.3 | 80.2 | 85.2 | 84.0 | 79.5 |
| 20 | MI | 33. What are effective methods to prevent mental health and psychosocial problems/ promote mental health and psychosocial wellbeing in humanitarian settings? | 82.7 | 70.4 | 85.2 | 81.5 | 76.5 | 79.3 |
| 21 | RI | 68. What are appropriate ethical standards in the delivery of and research concerning mental health and psychosocial support in humanitarian settings? | 80.2 | 70.4 | 77.8 | 80.2 | 87.7 | 79.3 |
| 22 | PA | 6. What are the major risk factors (including individual [e.g. age, gender] and contextual [e.g. poverty, domestic violence]) for mental health and psychosocial problems in humanitarian settings? | 81.5 | 76.5 | 76.5 | 80.2 | 81.5 | 79.2 |
| 23 | MC | 46. What are locally perceived needs for mental health and psychosocial support in humanitarian settings? | 80.2 | 82.7 | 74.1 | 76.5 | 81.5 | 79.0 |
| 24 | MI | 32. What is the effectiveness of integrating mental health and psychosocial considerations in other sectors/ clusters (nutrition, shelter, hygiene and sanitation) to prevent mental disorders, protect psychosocial wellbeing, and promote mental health and psychosocial wellbeing? | 87.7 | 64.2 | 77.8 | 80.2 | 81.5 | 78.3 |
| 25 | MC | 51. How can existing strengths and capacities of communities best be integrated in existing or newly developed mental health and psychosocial supports? | 82.7 | 69.1 | 80.2 | 77.8 | 81.5 | 78.3 |
| 26 | PA | 10. What are women's experiences of mental health and psychosocial wellbeing? | 72.8 | 84.0 | 74.1 | 79.0 | 80.2 | 78.0 |
| 27 | MC | 53. How is the coordination of mental health and psychosocial support in humanitarian settings currently organized and how could this be improved? | 80.2 | 69.1 | 79.0 | 79.0 | 82.7 | 78.0 |
| 28 | MI | 22. What is the effectiveness of targeting mother's mental health and psychosocial problems to prevent mental disorders and protect and promote psychosocial wellbeing and mental health among children and adolescent in humanitarian settings? | 81.5 | 74.1 | 76.5 | 79.0 | 77.8 | 77.8 |
| 29 | MI | 38. How can mental health and psychosocial supports in humanitarian settings best be adapted in order to increase accessability for people with special needs (e.g. children and adults with disabilities, orphans)? | 76.5 | 70.4 | 74.1 | 86.4 | 81.5 | 77.8 |
| 30 | MI | 30. What is the effectiveness of including mental health and psychosocial components in disaster preparedness activities to prevent mental disorders and protect and promote psychosocial wellbeing and mental health? | 82.7 | 58.0 | 81.5 | 84.0 | 81.5 | 77.5 |
| 31 | MI | 39. Which interventions should be combined in a core package of mental health and psychosocial support in humanitarian settings? | 84.0 | 66.7 | 81.5 | 76.5 | 79.0 | 77.5 |
| 32 | MC | 49. In what ways do communities respond to address the mental health and psychosocial impact of humanitarian crises? | 77.8 | 71.6 | 79.0 | 75.3 | 82.7 | 77.3 |
| 33 | MI | 29. What is the cost-effectiveness of integrating mental health care in primary care settings in humanitarian settings? | 86.4 | 70.4 | 76.5 | 75.3 | 76.5 | 77.0 |
| 34 | PA | 8. Which factors determine resilience for individuals and communities in humanitarian settings? | 87.7 | 58.0 | 74.1 | 77.8 | 81.5 | 75.8 |
| 35 | MI | 34. What is the effectiveness of Psychological First Aid to prevent longer-term mental health and psychosocial consequences in acute phases of humanitarian crises? | 81.5 | 66.7 | 80.2 | 77.8 | 72.8 | 75.8 |
| 36 | MI | 23. What is the effectiveness of recreational activities (e.g. sports, youth club activities) to prevent mental disorders and protect and promote psychosocial wellbeing and mental health among children and adolescent in humanitarian settings? | 72.8 | 72.8 | 77.8 | 72.8 | 82.7 | 75.8 |
| 37 | RI | 74. How can research best be integrated in mental health and psychosocial support programs in humanitarian settings? | 80.2 | 67.9 | 75.3 | 75.3 | 77.8 | 75.3 |
| 38 | MC | 52. What are contextual barriers and facilitators of succesful delivery of mental health and psychosocial support in humanitarian settings? | 79.0 | 70.4 | 75.3 | 75.3 | 76.5 | 75.3 |
| 39 | PA | 12. What do affected populations view as causes of mental health and psychosocial problems in humanitarian settings? | 76.5 | 80.2 | 69.1 | 70.4 | 80.2 | 75.3 |
| 40 | MI | 24. What is the effectiveness of community mobilization to prevent mental disorders and protect and promote psychosocial wellbeing and mental health in humanitarian settings? | 86.4 | 55.6 | 76.5 | 80.2 | 76.5 | 75.0 |
| 41 | RI | 72. What are appropriate methods to adapt existing instruments to assess, monitor, and evaluate mentalhealth and psychosocial wellbeing in humanitarian settings? | 74.1 | 76.5 | 70.4 | 74.1 | 75.3 | 74.1 |
| 42 | MI | 28. What is the effectiveness of rehabilitation efforts for people with severe mental disorders in humanitarian settings? | 74.1 | 67.9 | 70.4 | 79.0 | 77.8 | 73.8 |
| 43 | MI | 19. What is the effectiveness of educational interventions (e.g. formal and non-formal education) to prevent mental disorders and protect and promote psychosocial wellbeing and mental health among children and adolescent in humanitarian settings? | 82.7 | 63.0 | 72.8 | 74.1 | 74.1 | 73.3 |
| 44 | MC | 63. What is the effectiveness of the implementation of the IASC Guidelines on Mental Health and Psychosocial Support in Emergencies? | 85.2 | 58.0 | 71.6 | 72.8 | 79.0 | 73.3 |
| 45 | MC | 45. What is the effectiveness of task shifting, i.e. delivery of specialized mental health and psychosocial support by staff with less specialized training? | 79.0 | 71.6 | 76.5 | 75.3 | 63.0 | 73.1 |
| 46 | MC | 41. What are the long-term benefits of mental health and psychosocial support in humanitarian settings? | 84.0 | 50.6 | 72.8 | 81.5 | 75.3 | 72.8 |
| 47 | MC | 43. What are the possible negative outcomes of mental health and psychosocial support in humanitarian settings? | 77.8 | 63.0 | 77.8 | 74.1 | 70.4 | 72.6 |
| 48 | MC | 48. What are local perceptions towards people (including misperceptions) with mental disorders and psychosocial problems in humanitarian settings, and how are they treated? | 67.9 | 79.0 | 67.9 | 70.4 | 72.8 | 71.6 |
| 49 | MI | 18. What is the effectiveness of child-friendly spaces to prevent mental disorders and protect and promote psychosocial wellbeing and mental health among children and adolescent in humanitarian settings? | 72.8 | 67.9 | 70.4 | 71.6 | 72.8 | 71.1 |
| 50 | MI | 21. What is the effectiveness of preventing child-family seperation and family reunion to prevent mental disorders and protect and promote psychosocial wellbeing and mental health among children and adolescent in humanitarian settings? | 76.5 | 60.5 | 67.9 | 79.0 | 70.4 | 70.9 |
| 51 | RI | 73. What are appropriate methods to develop new instruments to assess, monitor, and evaluate mentalhealth and psychosocial wellbeing in humanitarian settings? | 75.3 | 70.4 | 70.4 | 65.4 | 69.1 | 70.1 |
| 52 | MC | 57. How does the type of humanitarian setting (e.g. political violence and natural disasters) impact delivery of mental health and psychosocial support? | 72.8 | 63.0 | 70.4 | 69.1 | 74.1 | 69.9 |
| 53 | MC | 60. What is the impact of humanitarian crises on the mental health and psychosocial wellbeing of people delivering mental health and psychosocial support? | 63.0 | 79.0 | 75.3 | 61.7 | 70.4 | 69.9 |
| 54 | MC | 64. How can the IASC Guidelines best be applied operationally, beyond the stated principles? | 80.2 | 56.8 | 71.6 | 67.9 | 72.8 | 69.9 |
| 55 | PA | 4. Which mental health and psychosocial problems in the acute phase of humanitarian settings predict problems on the longer term? | 80.2 | 54.3 | 71.6 | 75.3 | 66.7 | 69.6 |
| 56 | MC | 44. What is the appropriate timing/ sequencing of mental health and psychosocial supports in humanitarian settings? | 79.0 | 54.3 | 72.8 | 71.6 | 69.1 | 69.4 |
| 57 | RI | 69. What are appropriate methods to screen populations for inclusion in mental health and psychosocial support in humanitarian settings? | 74.1 | 65.4 | 67.9 | 66.7 | 69.1 | 68.6 |
| 58 | MI | 26. What other community-based efforts may be effective in preventing mental disorders and protecting and promoting psychosocial wellbeing and mental health in humanitarian settings? | 75.3 | 59.3 | 67.9 | 67.9 | 71.6 | 68.4 |
| 59 | MI | 37. What is the efficacy/ effectiveness of stress management and self-care workshops to prevent mental disorders, protect psychosocial wellbeing, and promote mental health and psychosocial wellbeing in humanitarian settings? | 66.7 | 69.1 | 74.1 | 61.7 | 67.9 | 67.9 |
| 60 | MC | 59. What are the minimum financial requirements to implement a core package of mental health and psychosocial support in humanitarian settings? | 76.5 | 55.6 | 66.7 | 70.4 | 69.1 | 67.7 |
| 61 | MC | 58. How do global and local financing mechanisms impact the delivery of mental health and psychosocial support in humanitarian settings? | 71.6 | 59.3 | 63.0 | 67.9 | 72.8 | 66.9 |
| 62 | MI | 35. What is the effectiveness of psychosocial counselling to treat mental health and psychosocial problems to prevent mental disorders, protect psychosocial wellbeing, and promote mental health and psychosocial wellbeing in humanitarian settings? | 70.4 | 61.7 | 69.1 | 64.2 | 66.7 | 66.4 |
| 63 | MI | 27. What types of psychiatric medication should be included in an essential drug package in humanitarian settings? | 63.0 | 74.1 | 70.4 | 60.5 | 63.0 | 66.2 |
| 64 | MI | 31. What is the effectiveness of integrating mental health and psychosocial considerations in human rights programs to prevent mental disorders, protect psychosocial wellbeing, and promote mental health and psychosocial wellbeing? | 75.3 | 49.4 | 60.5 | 76.5 | 69.1 | 66.2 |
| 65 | MC | 56. How could technology (including internet, film, telephone) assist in the delivery of mental health and psychosocial support in humanitarian settings? | 64.2 | 69.1 | 65.4 | 60.5 | 67.9 | 65.4 |
| 66 | MI | 20. What is the effectiveness of psychosocial stimulation activities for young children to prevent mental disorders and protect and promote psychosocial wellbeing and mental health among young children in humanitarian settings? | 65.4 | 61.7 | 61.7 | 64.2 | 67.9 | 64.2 |
| 67 | MC | 61. What are effective ways to select staff for the delivery of mental health and psychosocial support in humanitarian settings? | 69.1 | 59.3 | 63.0 | 59.3 | 69.1 | 64.0 |
| 68 | RI | 67. How can we distinguish between 'normal' and 'pathological' mental health and psychosocial conditions in humanitarian settings? | 65.4 | 61.7 | 61.7 | 65.4 | 64.2 | 63.7 |
| 69 | MC | 40. How could a gender analysis assist in maximizing the impact of mental health and psychosocial support in humanitarian settings? | 58.0 | 58.0 | 58.0 | 72.8 | 71.6 | 63.7 |
| 70 | RI | 66. To what extent do disagreements in definitions of key concepts (e.g. psychosocial wellbeing, trauma) impact humanitarian practice? | 56.8 | 56.8 | 60.5 | 63.0 | 71.6 | 61.7 |
| 71 | MC | 54. What type of organizations are best positioned to deliver succesful mental health and psychosocial support in humanitarian settings? | 64.2 | 49.4 | 59.3 | 63.0 | 61.7 | 59.5 |
| 72 | MI | 25. What is the effectiveness of traditional and cultural healing practices (e.g. exorcism of evil spirits, rituals aimed at cleansing or forgiveness, meditation practices) to prevent mental disorders and protect and promote psychosocial wellbeing and mental health in humanitarian settings? | 70.4 | 49.4 | 59.3 | 58.0 | 53.1 | 58.0 |
| 73 | PA | 9. How do biological, psychological, and social factors interact in the long term to influence mental health and psychosocial wellbeing in humanitarian settings? | 72.8 | 28.4 | 56.8 | 59.3 | 64.2 | 56.3 |
| 74 | RI | 65. Are there universal definitions and diagnostic criteria for mental health and psychosocial problems in humanitarian settings? | 46.9 | 45.7 | 43.2 | 46.9 | 54.3 | 47.4 |

Notes: PA = Problem Analysis; MI = Mental health and psychosocial support Interventions; MC = Mental health and psychosocial support Context; RI = Research Issues
